# Shoulder Pain Secondary to Mediastinal Myelolipoma: A Case Report

**DOI:** 10.7759/cureus.86579

**Published:** 2025-06-23

**Authors:** Govini Balasubramani, Sindhura Koganti, Sneha Badhey, Isha Govini

**Affiliations:** 1 Heart and Lung Transplant, Gleneagles Hospitals, Chennai, IND; 2 Lung Transplant, Gleneagles Hospitals, Chennai, IND; 3 Internal Medicine, Texas Health Resources, Denton, USA; 4 Pulmonology, Chettinad Hospitals, Chennai, IND

**Keywords:** benign, extra-adrenal, incidental mediastinal mass, intrathoracic mass, myelolipoma

## Abstract

Myelolipomas are tumors composed of fat and hematopoietic elements. Typically, these neoplasms are benign and found in the adrenal glands. In certain occurrences, however, they can be found in other physiologic regions. Rarely do these become symptomatic, which may also contribute to the limited instances noted in medical literature. In this report, we outline an incidentally found extra-adrenal myelolipoma in an otherwise healthy patient, along with the subsequent diagnostic and treatment modalities employed. Given the rarity of such cases, this report aims to contribute to the existing literature and help familiarize and guide healthcare professionals in the diagnosis and management of these conditions.

## Introduction

Myelolipomas are predominantly benign neoplasms consisting of adipose and hematopoietic cells. In the classification of myelolipomas, there are typically four patterns noted in clinical practice: (1) isolated adrenal myelolipomas, (2) hemorrhagic adrenal myelolipomas, (3) myelolipomas associated with adrenal pathology, and in rare instances, (4) extra-adrenal myelolipomas [1]. Most commonly, myelolipomas are found in the adrenal glands, and associated complications can include overproduction of adrenal hormones or compression of structures in the retroperitoneal region. The incidence of myelolipomas is estimated to be less than 1% based on autopsy studies. However, among those, extra-adrenal cases are estimated to account for approximately 15% [2]. These have been noted to be more prevalent in adults between the ages of 50 and 70 years old, without a gender predilection [3]. Extra-adrenal cases are typically rare, as their discovery can either be incidental or, in more concerning cases, if they affect the functionality of surrounding structures. In the following report, we outline a case of an incidentally found mediastinal myelolipoma. We also discuss potential consequences that may occur in such a case of an undetected neoplasm, especially regarding anatomical location. We also suggest modalities of surgical and medical management to facilitate earlier detection and management of such cases, thereby improving overall outcomes and preventing complications that may arise from an undetected neoplasm of this kind.

## Case presentation

A 54-year-old nonsmoking male presented for a routine wellness examination with mild right-sided shoulder pain. The patient's past medical history was noted to be relatively unremarkable. Apart from being slightly overweight, he had no known chronic medical conditions and did not take any daily medications. The patient worked as a farmer and did not report any unusual chemical exposures in his day-to-day life. He also denied any known physical trauma prior to the onset of his symptoms. In his initial presentation, he reported right-sided shoulder pain for the past year, which had been worsening over the past two months. Initially, the pain was characterized as a dull ache; however, over the preceding few months, the ache had become more noticeable and constant. Shoulder pain was noted to be worse with exertion and better with rest. He also reported intermittent chest tightness during episodes of pain. Chest tightness was associated with what he described as "heavy breathing," or a midsternal pressure, especially while lying flat. He specifically denied experiencing chest pain, arm numbness or tingling, vision changes, hearing changes, fever, or night sweats. The physical exam revealed a male who appeared to be his stated age and was in no acute distress.

**Figure 1:**
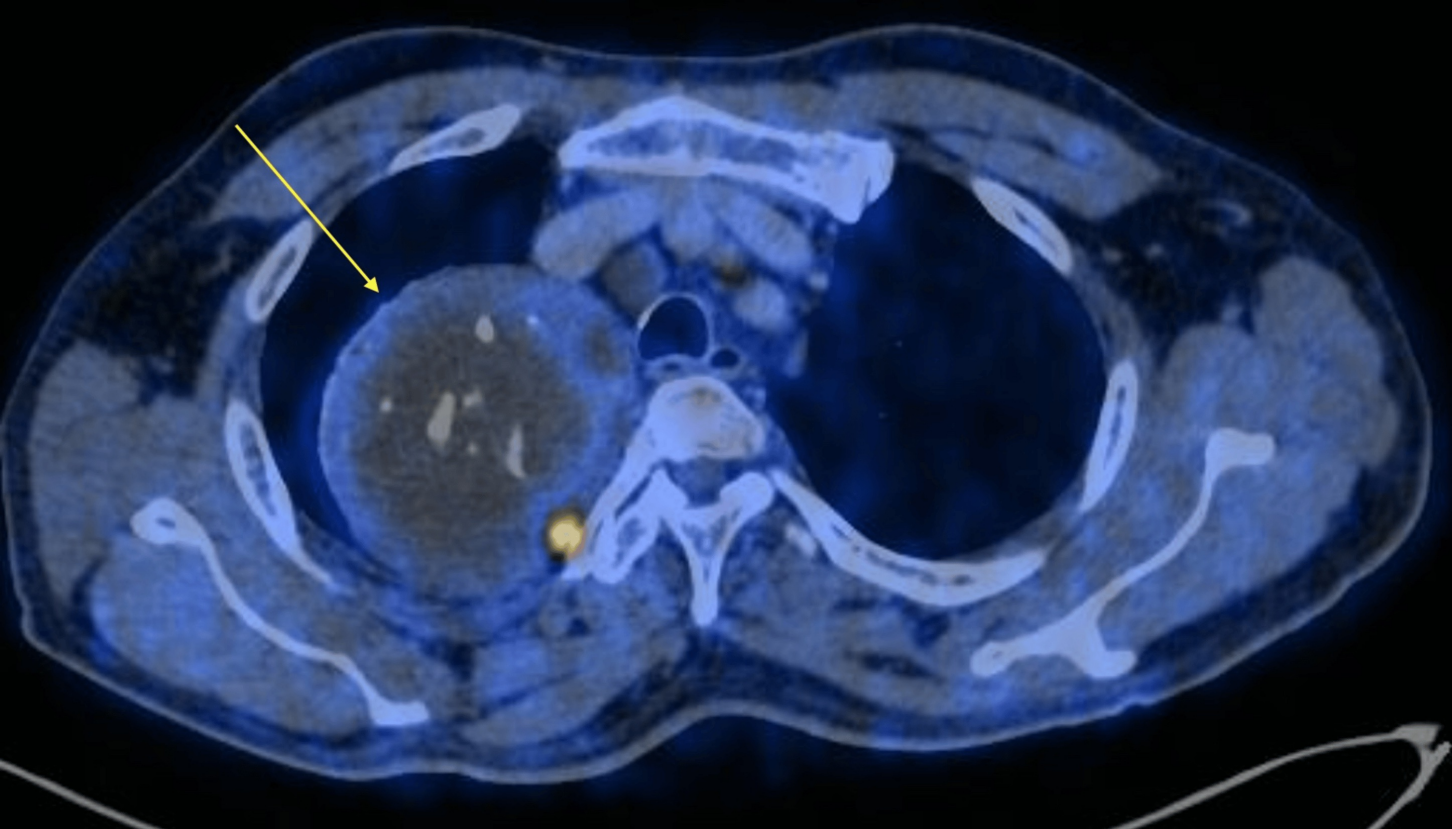
Yellow arrow indicates the 9.5 cm x 10 centimeter neoplasm in the right upper hemithorax PET: positron emission tomography

Apart from being mildly overweight, vital signs were unremarkable. The cardiovascular exam did not reveal any murmur, extra heart sounds, friction rub, or appreciable arrhythmias. His pulmonary exam was also unremarkable, without any wheezes, crackles, or rales. Additionally, range of motion, specifically in his right upper extremity, was intact, and pain was not reproducible with passive or active manipulation. Initial laboratory studies, including a complete blood count and a comprehensive metabolic panel, did not reveal any significant abnormalities. For further evaluation of chest tightness, a chest x-ray was ordered, which incidentally revealed a mediastinal mass. For further classification, a positron emission tomography (PET) scan was done, showing a large, well-defined, pleural-based lesion with focally increased peripheral metabolic activity and coarse calcification and focal fat density in the right upper hemithorax (Figure 1).

**Figure 2:**
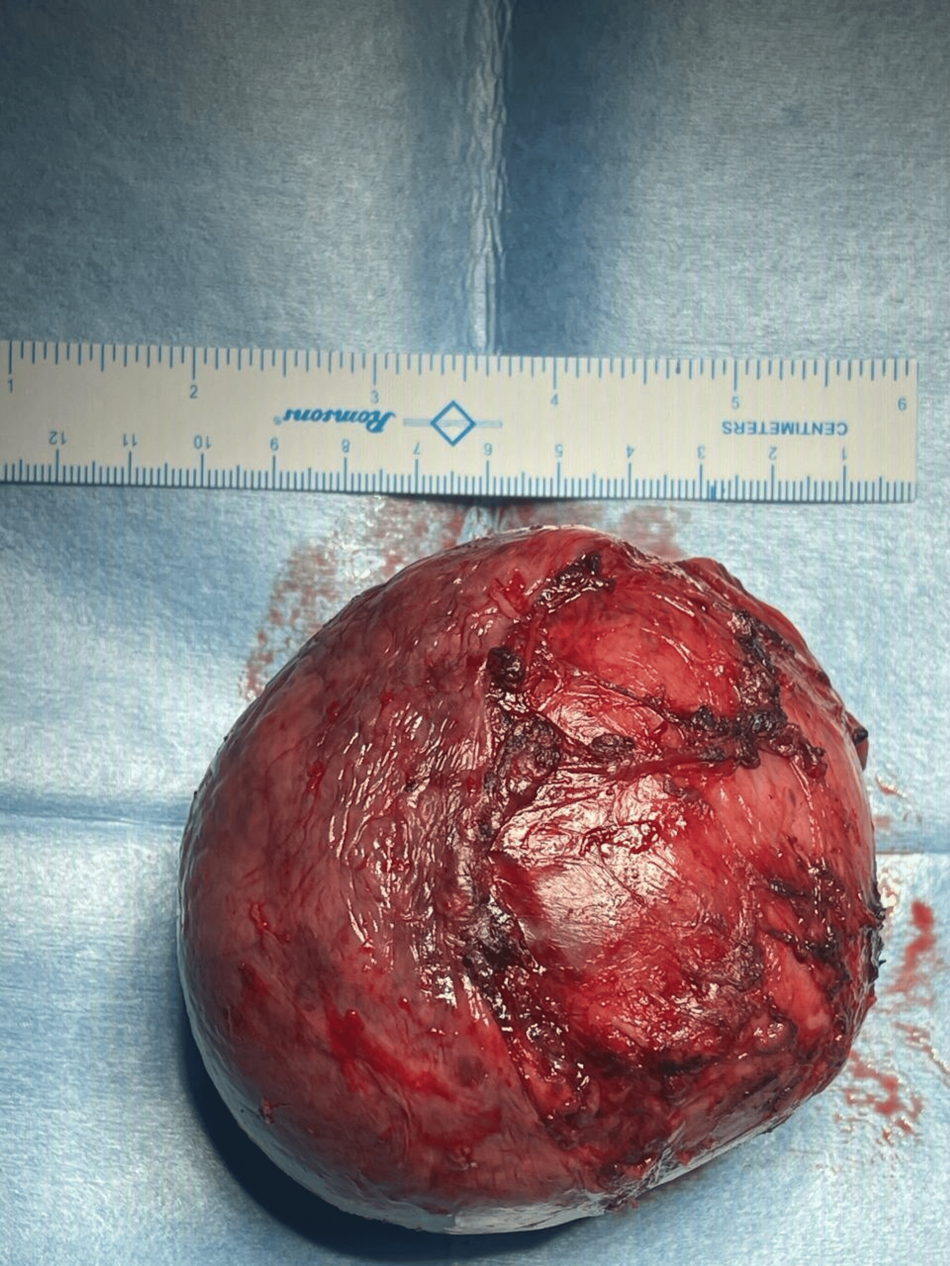
Status post excision of mediastinal mass

The lesion was noted to be abutting the mediastinal pleura medially and the right upper lobar bronchus inferiorly. It did not reveal any fluorodeoxyglucose uptake. The patient was referred to the thoracic surgery department. Given that the tumor burden did not affect any significant vasculature for which an exploratory surgery was indicated, video-assisted thoracoscopic surgery, during which the biopsy would be done, was the chosen method of excision. This was the preferred method, given that excision of the mass was indicated to improve the patient's overall complaints. Furthermore, this was also chosen to enhance postoperative recovery time and reduce mobility impairment. The patient tolerated excision and biopsy of the mass (Figure 2) without any significant complications.

**Figure 3:**
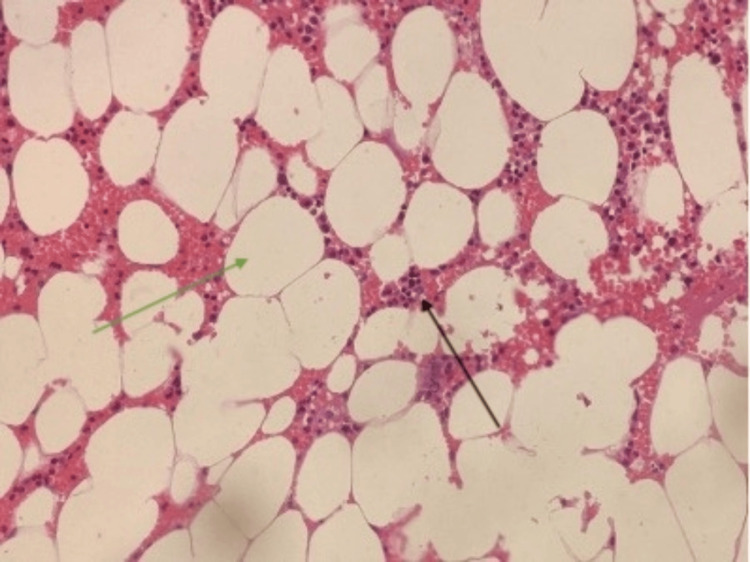
Pathology slide visualizing adipocytes (green arrow) with interspersed hematopoietic elements (black arrow)

Pathology reports revealed a lipoma of benign etiology (Figure 3).

The postoperative and recovery process was unremarkable, and the patient was revisited for a follow-up one week post-hospital discharge. He was deemed to be in stable condition and reported resolving the pain in his shoulder.

## Discussion

The above case describes a rare finding of an adrenal myelolipoma in an unexpected location. While myelolipomas are generally benign, they are typically associated with the adrenal glands. In fact, after adenomas, myelolipomas are the second most common primary adrenal incidentaloma [4]. Consistent with their nomenclature, myelolipomas consist of adipose tissue and hematopoietic cells. The etiology of these masses, as outlined in the current scientific literature, has several postulations. Convincing evidence notes that those patients with elevated levels of adrenocorticotropic hormone, such as in Cushing's disease or congenital adrenal hyperplasia, have been incidentally found to have myelolipomas [5]. Additional etiologies include abnormalities noted with bone marrow element production and circulation [6]. In terms of extra-adrenal myelolipomas, however, the causes are less clear.

Suspected causes include the activation of connective tissue hematopoiesis, which is typically observed in embryonic studies; however, definitive studies are still needed [7]. Extra-adrenal cases are extremely rare. Most commonly, extra-adrenal myelolipomas are found in the renal pelvis or retroperitoneal cavity [8]. Retroperitoneal myelolipomas have seen more traction in medical literature and do have increased potential to become symptomatic. For example, a case reported in the Archives of Pathology and Laboratory Medicine in 2006 outlines a 65-year-old male presenting with flank pain, hematuria, dysuria, and weight loss. Further imaging revealed a suspicious lung nodule, prompting a further workup that identified a myelolipoma in the right renal pelvis [9]. Those found in the thoracic cavity tend to have less medical visibility.

When a mass is found in the thoracic cavity, the typical differential diagnoses include, but are not limited to, thymomas, vascular deformations, thyroid tumors, lung tumors, and even lymphadenopathy in the setting of malignancy. Myelolipomas, especially in the thoracic cavity, are not usually the primary suspected neoplasms. However, the more benign nature of these tumors makes their presence relatively more favorable. Due to this, routine screening or detection is typically not indicated. It must be noted that detection of these masses often requires nonspecific symptoms or incidental imaging findings. Depending on location, a mass in the relatively larger thoracic cavity may require a longer period of growth before symptoms arise. Thus, fewer are likely to cause symptoms until the mass size necessitates expansion. In the above case, the patient likely had a mass effect due to the increasing size of thoracic structures and nerves innervating the upper extremity, prompting further workup. This can be supported by the reported alleviation of symptoms after excision. However, avoidance of removal may have caused increased growth of said mass, leading to vascular stenoses/occlusions, respiratory distress due to compression of pulmonary structures, nerve damage due to chronic compression, and even cardiac complications depending on the size. Hormone studies should be considered in cases of abnormal vital signs or persistent electrolyte imbalances. In those in whom conservative management is pursued, for example, if the risk of surgery outweighs the benefit, routine imaging at provider-determined intervals (with either x-ray or computed tomography scans) may be indicated, depending on location and risk to surrounding organs. The risks of extra-adrenal myelolipomas will again depend on their location. Adrenal myelolipomas have been found to carry the risk of hemorrhage, especially when size exceeds 4 centimeters; however, the same statistic has not been routinely extrapolated to extra-adrenal myelolipomas [10].

Management of extra-adrenal myelolipomas should involve considerations of size, pertinent symptoms, and associated derangements (if any) in metabolic and hormone panels. Comprehensive clinical judgment must be exercised regarding the surveillance and treatment of these cases. Healthcare personnel should carefully consider the risks and benefits of each management modality when caring for a patient with an extra-adrenal myelolipoma. Specialist evaluation and input are almost always necessary. We present this case here to not only contribute to the current literature but also acknowledge that the existing data on this topic is limited. We hope to increase awareness of this rare finding and encourage healthcare providers' vigilance and understanding of its management. We acknowledge and strongly advocate for further studies in this field to develop standardized guidelines for the timely diagnosis and management of extra-adrenal myelolipomas.

## Conclusions

This case of an extra-adrenal myelolipoma illustrates the variability of tumor presentation, as evidenced by the peculiar presentation of this otherwise healthy patient. While by themselves they are typically benign, extra-adrenal myelolipomas can manifest with abnormal symptom presentation and indirectly cause consequences secondary to mass effect. In patients with an unidentified mass, careful examination, specialist intervention, and comprehensive evaluation are necessary for accurate identification and subsequent management to achieve optimal outcomes.
